# Association between dietary zinc intake and epilepsy: findings from NHANES 2013–2018 and a Mendelian randomization study

**DOI:** 10.3389/fnut.2024.1389338

**Published:** 2024-07-10

**Authors:** Shicun Huang, Ya Gao, Yingqi Chen, Yiqing Wang, Yeting Lu, Wei Gao, Xiaowei Hu, Qi Fang

**Affiliations:** ^1^Department of Neurology, First Affiliated Hospital of Soochow University, Suzhou, China; ^2^Department of Neurology, Suzhou Guangci Cancer Hospital, Suzhou, China; ^3^Department of Neurology, Suzhou Hospital of Traditional Chinese Medicine, Suzhou, China

**Keywords:** zinc, epilepsy, NHANES, cross-sectional study, Mendelian randomization

## Abstract

**Background:**

The association between dietary zinc intake and epilepsy remains unclear. This study aimed to investigate the relationship between zinc intake from the diet and epilepsy, employing Mendelian randomization (MR) to explore potential causal links between zinc and epilepsy.

**Methods:**

The cross-sectional study utilized data from the National Health and Nutrition Examination Survey (NHANES) conducted between 2013 and 2018. Among the 4,434 participants included, 1.5% (67/4,434) reported having epilepsy. Restricted cubic spline models and logistic regression models were employed to examine the relationships between dietary zinc intakes and epilepsy. Subsequently, a 2-sample Mendelian randomization (MR) analysis was conducted using the inverse variance weighted (IVW) approach as the primary analysis.

**Results:**

In the restricted cubic spline (RCS) analysis, the relationship between dietary zinc consumption and epilepsy displayed an L-shaped curve (nonlinear, *p* = 0.049). After multivariate adjustments, the adjusted odds ratios for epilepsy in T2 (5.0–11.0 mg/day) and T3 (≥11.0 mg/day) were 0.49 (95% confidence interval [CI]: 0.26–0.92, *p* = 0.026) and 0.60 (95% CI: 0.31–1.17, *p* = 0.132), respectively, compared to the lowest dietary zinc consumption tertile (T1, ≤5.0 mg/day). The IVW method indicated that genetically predicted zinc intake per standard-deviation increase was inversely associated with three types of epilepsy, including all types of epilepsy (OR = 1.06, 95% CI: 1.02–1.11, *p* = 0.008), generalized epilepsy (OR = 1.13, 95% CI: 1.01–1.25, *p* = 0.030), and focal epilepsy (documented hippocampal sclerosis) (OR = 1.01, 95% CI: 1.00–1.02, *p* = 0.025).

**Conclusion:**

Our findings suggest that a daily zinc intake ranging from 5.0 to 11.0 mg is associated with the lowest risk of epilepsy. Furthermore, Mendelian randomization (MR) studies provide additional support for the existence of a causal relationship between zinc and epilepsy.

## 1 Introduction

With an estimated 68 million cases worldwide, epilepsy stands as one of the most prevalent and serious chronic neurological disorders (1). Despite extensive research, the process of epileptogenesis, the emergence of epilepsy, remains poorly understood. Zinc has been implicated in several proposed theories to explain the pathophysiology of epilepsy (2).

From early neonatal brain development to the maintenance of adult brain function, the trace metal zinc is a biofactor crucial to the central nervous system. Zinc regulates synaptic activity and neural plasticity at the cellular level during both development and adulthood (3). At the molecular level, zinc controls the activity of numerous important enzymes involved in brain metabolism, as well as gene expression via transcription factor activity. Depending on the concentration levels in the CNS, zinc exhibits a biphasic response that can be both neurotoxic and neuroprotective (4). Research has emphasized zinc’s dual action in studying its impact on seizure latency and severity in rats modeling temporal lobe epilepsy. This was achieved through zinc supplementation alone or combined with valproic acid pre-treatment, a common antiseizure medication (5).

Zinc might have a significant impact on the pathophysiology of epilepsy and/or seizures. Studies on zinc’s effects on seizures have revealed that zinc exhibits dose-dependent pro- and anticonvulsant properties (3, 6, 7). Zinc may be neurotoxic in large quantities; however, in animal models, zinc at moderate concentrations has been demonstrated to enhance the anti-epileptic effects of conventional medications (8). Despite the diverse roles zinc plays in the central nervous system, one term encapsulating all its multiple effects is “homeostasis” (3). Zinc homeostasis can be altered by an excessive or insufficient zinc intake, leading to malfunctioning cellular systems. Therefore, moderate zinc intake through diet can work in conjunction with conventional antiseizure medication (4).

There is a paucity of studies related to dietary zinc intake in people with epilepsy and there is a lack of clarity about the levels of zinc ingested. Utilizing information from the National Health and Nutrition Examination Survey (NHANES), we conducted a cross-sectional study to investigate the relationship between dietary zinc intake and epilepsy. Previous studies have also not assessed the causal association between the two. Genetic variations are used as instrumental variables in genetic Mendelian randomization (MR) analysis, a technique that examines the relationship between disease phenotypes and clinical features (9). To control for confounders and reverse causation, MR is preferable to observational research. This is because genetic alleles are randomly assigned during meiosis and are not influenced by environmental factors (10). Therefore, we also conducted a concurrent MR study to determine the causal association between the two.

## 2 Methods

### 2.1 Overall study design

The current investigation comprised two sections. In the initial section, we examined the association between dietary zinc intake levels and epilepsy using data from the NHANES, while controlling for various potential confounders. In the subsequent section, we utilized MR analysis of summary statistics from a genome-wide association study (GWAS) to assess the causal relationship between genetically predicted zinc levels and epilepsy.

### 2.2 Observational study

#### 2.2.1 Study population in NHANES

**Figure 1:**
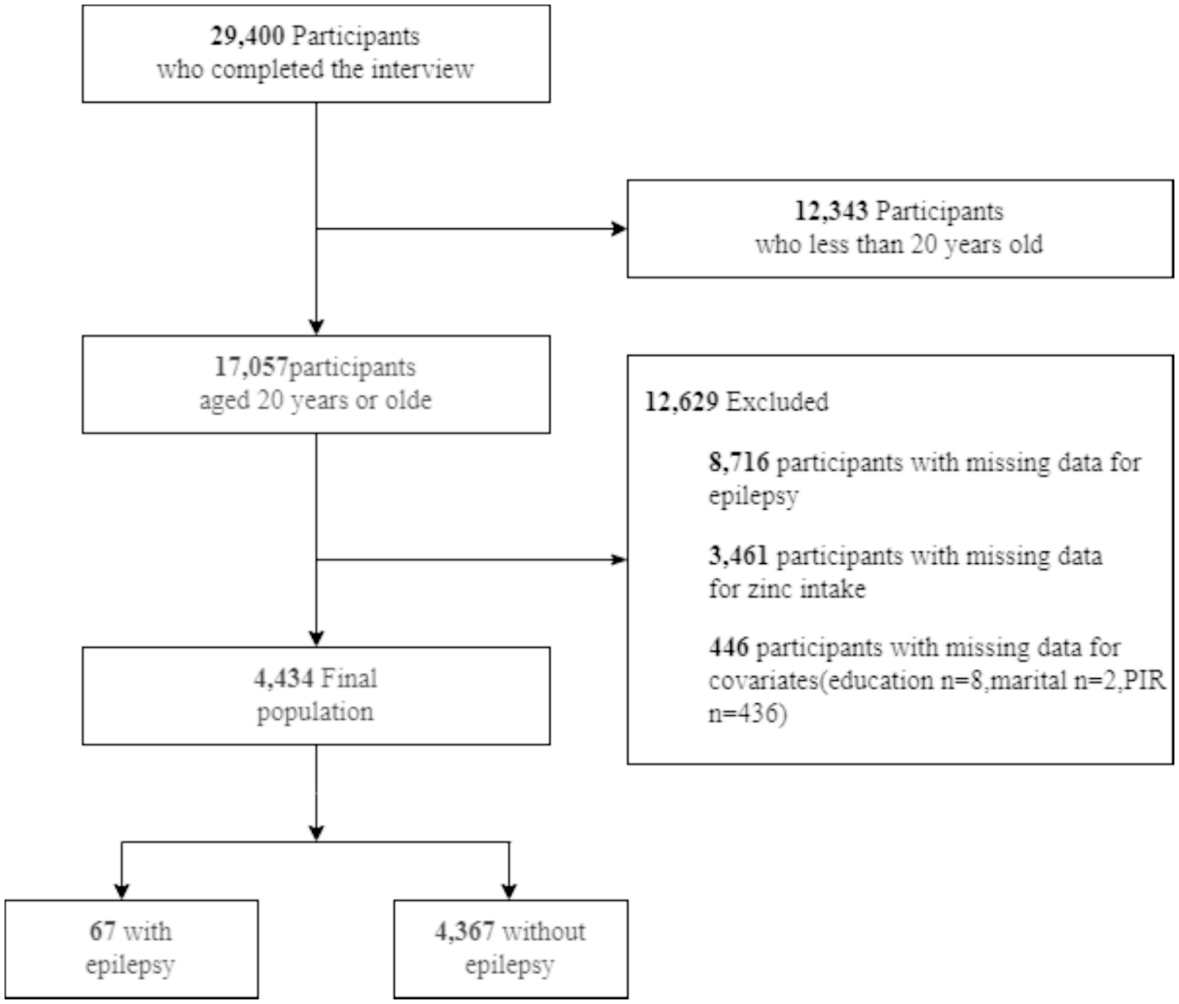
The study’s flow diagram.

The Centers for Disease Control and Prevention conducts NHANES, an annual cross-sectional survey involving approximately 5,000 Americans. The survey covers demographics, nutrition, examination, laboratory, and questionnaire areas (11). The study adhered to the Declaration of Helsinki, obtaining written consent from participants. Professionals conducted investigations, including blood tests in mobile centers and interviews at participants’ homes. NHANES data can be accessed on their website^1^ as of December 1, 2023. We analyzed NHANES data from three cycles (2013–2014, 2015–2016, and 2017–2018) regarding epilepsy and diet. Preliminary data excluded 12,343 minors among 29,400 respondents. Among 17,057 adults, 8,716 lacked sufficient data on zinc intake, and 3,461 lacked sufficient data on epilepsy. An additional 446 participants were disqualified due to incomplete demographic information. Ultimately, 67 out of 4,434 individuals with complete data had an official epilepsy diagnosis (Figure 1).

#### 2.2.2 Diagnosis of epilepsy

Data on epilepsy were collected through face-to-face interactions between the subjects and the researchers. During the survey, respondents were asked to list any prescription drugs they had taken in the previous 30 days under medical supervision, along with a detailed explanation of the reasons for their use. If a medication reported for seizures was not to be an Anti-Seizure Medication (ASM) upon manual review, we excluded such medications from our case definition (Supplementary Table S1). The International Classification of Diseases (ICD) code for “epilepsy and recurrent seizures” (G40) was used to categorize participants, and those on medication were considered to be epilepsy patients.

#### 2.2.3 Dietary zinc intake

The NHANES dietary survey, conducted between 2013 and 2018, assessed participants’ food and drink intake over a 24 h period. The Automated Multiple Pass Method by the United States Department of Agriculture was employed for data collection (12). These data enabled precise nutrient calculations for participants based on their dietary choices. While 24 h dietary recalls have inherent limitations, they offer more detailed information on food types and quantities compared to food frequency questionnaires (13, 14). Patients were stratified into three groups according to their zinc intake levels: T1 group (<5.0 mg per day), T2 group (5.0–11.0 mg per day), and T3 group (>11.0 mg per day). Detailed methodologies are outlined in the NHANES Dietary Interviewers Procedure Manuals (11).

#### 2.2.4 Covariates

A variety of potential covariates were assessed according to previous studies (15–18), including age, sex, marital status, race/ethnicity, education level, family income, and dietary supplements taken. In the investigation of the association between dietary zinc consumption and epilepsy, all relevant covariates were considered potential confounders. Participants were categorized into two age groups: 20–50 and >50 years old. Race and ethnicity were classified into Mexican Americans, non-Hispanic Whites, non-Hispanic Blacks, and other races. Marital status had three categories: married, living with a partner, and living alone. Educational achievement was divided into three ranges: less than nine years, nine to twelve years, and more than twelve years. According to a US government report (12), the poverty income ratio (PIR) was used to divide household income into three categories: low (PIR ≤ 1.3), medium (PIR > 1.3 to 3.5), and high (PIR > 3.5). The question about medications and nutritional supplements taken during the previous month was used to calculate dietary supplements. This study followed the Strengthening the Reporting of Observational Studies in Epidemiology (STROBE) reporting guideline (19).

#### 2.2.5 Statistical analysis

NHANES aimed to collect data representative of the noninstitutionalized civilian population in the United States. Continuous variables in the characteristics of the study population are reported as the mean (standard deviation, SD) or proportions (%). Participants were stratified into tertiles based on their dietary zinc intake concentrations, with the lowest level assigned as the reference group (tertile 1, T1). The association between dietary zinc intake and epilepsy was examined through multivariable logistic regression analyses. Model 1 adjusted for age, sex, marital status, race/ethnicity, and education level. Model 2 incorporated family income and additional dietary supplements in addition to the variables in Model 1. Inflection points were determined using the likelihood-ratio test and bootstrap resampling method. Potential modifications to the relationship between dietary zinc and epilepsy were assessed for the following variables: sex, age (20–50 vs. >50 years), marital status (married or living alone vs. living with a partner), education level (≤12 years vs. >12 years), family income (low vs. medium or high), and dietary supplements. Subgroup heterogeneity was evaluated using multivariate logistic regression, and interactions between subgroups and dietary zinc intake were examined using likelihood ratio testing. Restricted cubic splines were employed to model the dose–response association between dietary zinc intake levels and epilepsy. R 4.3.2 (http://www.R-project.org, The R Foundation, Shanghai, China) (Accessed on December 1, 2023) and Free Statistics software (version 1.9; Beijing Free Clinical Medical Technology Co., Ltd.) (20) were the statistical software packages used for all analyses. A two-tailed test determined that a *p*-value of less than 0.05 was significant.

### 2.3 Mendelian randomization

#### 2.3.1 Study design

**Figure 2:**
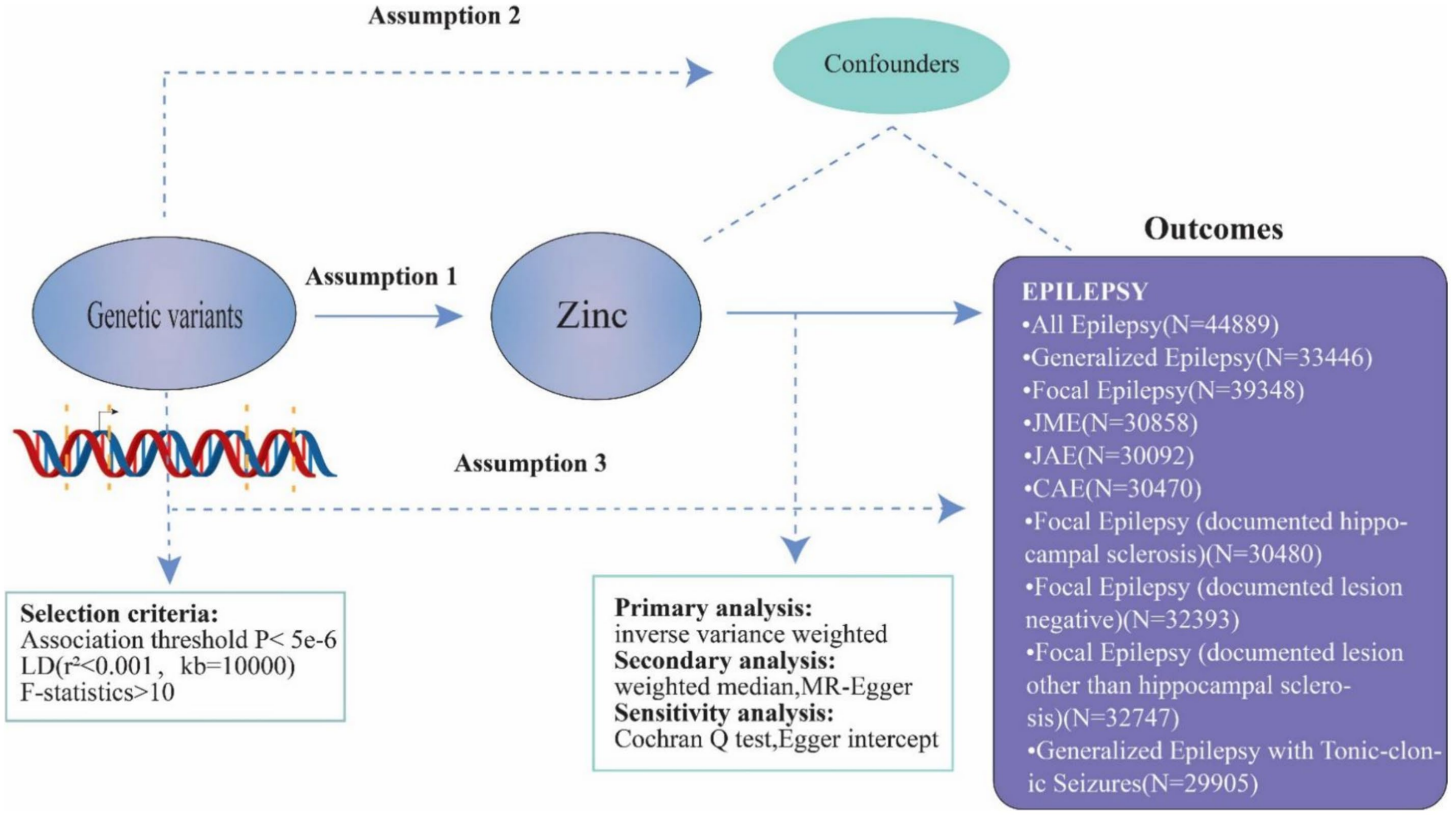
Principles of Mendelian randomization and assumptions. Principles of Mendelian randomization and assumptions. Assumption 1: exposure is robustly associated with genetic variants; Assumption 2: confounders are not associated with genetic variants; Assumption 3: genetic variants should influence the outcomes only mediated by the exposure of interest.

In order to establish the causative association between genetically predicted zinc levels and epilepsy in our investigation, we conducted a univariable two-sample MR analysis (Figure 2). To investigate the causal effects of exposure on the outcome, MR analysis must satisfy three assumptions: (1) genetic variants should be correlated with zinc levels; (2) these variants should not be associated with confounding factors; and (3) they should only influence epilepsy through the mediation of zinc levels.

#### 2.3.2 Genetic instrument selection

We integrated data from the GWAS for zinc, incorporating information from 2,603 European individuals (Supplementary Table S2). Our instrument selection process involved selecting single nucleotide polymorphisms (SNPs) with a genome-wide association with zinc levels (*p* < 5.0 × 10^−6^). Excluding SNPs in linkage disequilibrium (*r*^2^ threshold <0.001 within a 10,000 kb window), we extracted the remaining SNPs from the outcome datasets. Only SNPs with a minor Allele frequency (MAF) greater than 1% were considered in the study. *F*-statistics were calculated for each SNP to assess the strength of the exposures, excluding weak ones (*F*-statistics <10) (Supplementary Table S3) (21). The institutional review board approved and gave informed consent was obtained for all participants involved in this research. The study was conducted in compliance with STROBE-MR criteria (22).

#### 2.3.3 Outcome data

We combined pooled data from the GWAS encompassing various forms of epilepsy. The data for this study were provided by the International League Against Epilepsy (ILAE) Consortium cohort (23) (Supplementary Table S2).

#### 2.3.4 Statistical analysis

We utilized the inverse variance weighted (IVW) technique with random effects for obtaining causal estimates in the primary analysis. As secondary methods to address pleiotropy, we employed the weighted median (WM) method and MR-Egger regression. The Cochran *Q* test was used to assess the heterogeneity of certain SNPs, and a *p*-value of 0.05 from the Cochran *Q* indicated potential pleiotropy. To ascertain the balance of pleiotropy, we calculated the intercept using MR-Egger regression as a measure of directional pleiotropy (24, 25). The study and analysis were performed using R-version 4.3.2, utilizing the Mendelian Randomization and TwoSampleMR packages (26, 27).

## 3 Results

### 3.1 Sociodemographic characteristics

Table 1 presents the demographic information and health status of the participants. There were 1999 males, with a mean age of 59.0 (45.0, 70.0) years. A total of 67 individuals, accounting for 1.5% of the total, reported having epilepsy. Participants who were younger, male, married or cohabiting, non-Hispanic White, possessed higher educational levels, and had a medium family income tended to have a higher dietary zinc consumption.

**Table 1:** Population characteristics by categories of dietary zinc intake.

Characteristic	Zinc intake, mg/d				*p*-value
	Total	T1 (≤5.0)	T2 (5.0–11.0)	T3 (≥11.0)	
NO.	4,434	663	2,174	1,597	
Age (year), Mean (SD)	59.0 (45.0, 70.0)	61.0 (48.0, 71.0)	60.0 (45.0, 70.0)	57.0 (43.0, 68.0)	<0.001
Sex, *n* (%)					<0.001
Male	1999 (45.1)	187 (28.2)	878 (40.4)	934 (58.5)	
Female	2,435 (54.9)	476 (71.8)	1,296 (59.6)	663 (41.5)	
Race/ethnicity, *n* (%)					<0.001
Non-Hispanic white	2059 (46.4)	259 (39.1)	970 (44.6)	830 (52.0)	
Non-Hispanic black	971 (21.9)	214 (32.3)	474 (21.8)	283 (17.7)	
Mexican American	450 (10.1)	58 (8.7)	220 (10.1)	172 (10.8)	
Others	954 (21.5)	132 (19.9)	510 (23.5)	312 (19.5)	
Education level (year), *n* (%)					<0.001
<9	298 (6.7)	59 (8.9)	156 (7.2)	83 (5.2)	
9–12	1,487 (33.5)	255 (38.5)	708 (32.6)	524 (32.8)	
>12	2,649 (59.7)	349 (52.6)	1,310 (60.3)	990 (62.0)	
Marital status, *n* (%)					<0.001
Married or living with a partner	2,652 (59.8)	347 (52.3)	1,290 (59.3)	1,015 (63.6)	
Living alone	1782 (40.2)	316 (47.7)	884 (40.7)	582 (36.4)	
Family income, *n* (%)					<0.001
Low	1,186 (26.7)	236 (35.6)	584 (26.9)	366 (22.9)	
Medium	1,695 (38.2)	254 (38.3)	816 (37.5)	625 (39.1)	
High	1,553 (35.0)	173 (26.1)	774 (35.6)	606 (37.9)	
Dietary supplements taken, *n* (%)	2,811 (63.4)	406 (61.2)	1,377 (63.3)	1,028 (64.4)	0.370
Epilepsy, *n* (%)	67 (1.5)	17 (2.6)	26 (1.2)	24 (1.5)	0.041

### 3.2 Association between epilepsy and dietary zinc intake

As presented in Table 2, multivariable logistic regression models were employed to assess the association between dietary zinc intake and epilepsy. In Model 1, the risk of epilepsy in the second tertile (T2: 5.0–11.0 mg/day) and third tertile (T3: ≥11.0 mg/day) of dietary zinc consumption was 0.45 (95% CI: 0.24–0.84, *p* = 0.013) and 0.53 (95% CI: 0.27–1.02, *p* = 0.058), respectively, compared to the lowest tertile (T1: ≤5.0 mg/day). Following adjustment for all covariates, the odds ratios for epilepsy in T2 and T3 were 0.49 (95% CI: 0.26–0.92, *p* = 0.026) and 0.60 (95% CI: 0.31–1.17, *p* = 0.132), respectively, compared to the lowest tertile (T1: ≤5.0 mg/day). The risk of epilepsy was lowest with a daily consumption of 5.0–11.0 milligrams of zinc.

**Table 2:** Association between dietary zinc intake and epilepsy.

Tertiles	OR (95% CI)						
	No.	Crude	*p*-value	Model 1	*p*-value	Model 2	*p*-value
Dietary zinc (mg/day)							
T1(≤5.0)	663	1 (Ref)		1 (Ref)		1 (Ref)	
T2(5.0–11.0)	2,174	0.46 (0.25–0.85)	0.014	0.45 (0.24–0.84)	0.013	0.49 (0.26–0.92)	0.026
T3(≥11.0)	1,597	0.58 (0.31–1.09)	0.089	0.53 (0.27–1.02)	0.058	0.6 (0.31–1.17)	0.132
Trend test	4,434		0.200		0.129		0.235

T, tertiles; OR, odds ratio; CI, confidence interval; Ref, reference. Model 1 was adjusted for sociodemographic (age, sex, marital status, race/ethnicity, education level). Model 2 was adjusted for sociodemographic (age, sex, marital status, race/ethnicity, education level, family income) and dietary supplements taken.

### 3.3 Dose–response relationship analysis

**Figure 3:**
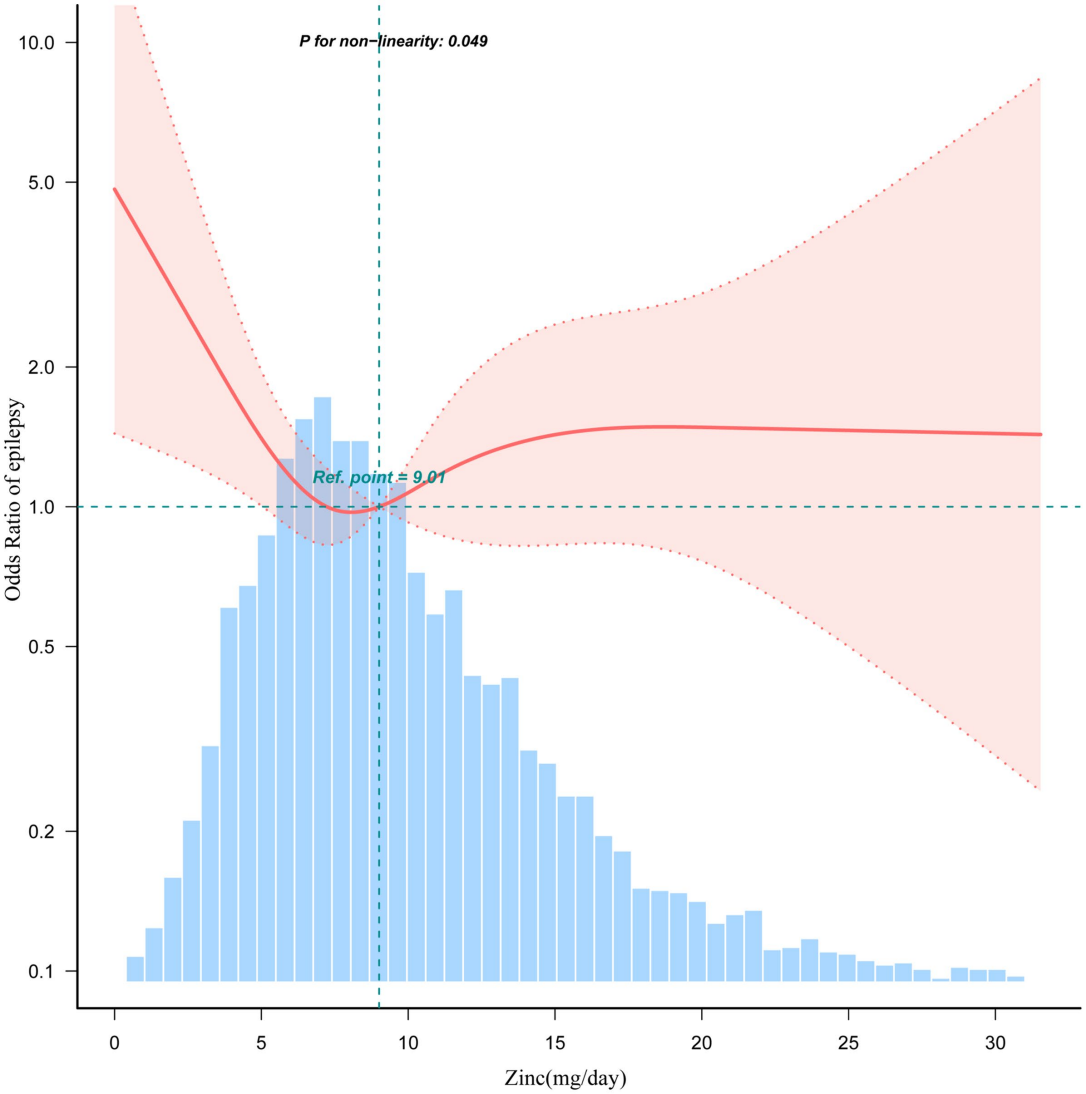
Association between dietary zinc intake and epilepsy odds ratio. Solid and dashed lines represent the predicted value and 95% confidence intervals. They were adjusted for sociodemographic (age, sex, marital status, race/ethnicity, education level, family income) and dietary supplements taken. Only 99% of the data is shown.

The association between dietary zinc intake and epilepsy revealed an L-shaped curve (nonlinear, *p* = 0.049) in RCS (Figure 3). In the threshold analysis, individuals consuming less than 8.0 mg of zinc daily had an OR of 0.81 (95% CI: 0.67–0.98, *p* = 0.031) for developing epilepsy (Supplementary Table S4). This suggests that beyond this threshold, increasing dietary zinc consumption is no longer associated with a decreased risk of epilepsy.

### 3.4 Stratified analyses based on additional variables

Stratified analyses were conducted in various subgroups to evaluate potential effect modifications in the relationship between dietary zinc and epilepsy. Subgroups included family income, sex, age, marital status, education level, and dietary supplements. No significant interactions were observed in any of the subgroups (Supplementary Table S5 and Supplementary Figure S1).

### 3.5 Mendelian randomization primary analysis and sensitivity assessment

The causal association between zinc and epilepsy was investigated through MR. In the initial stage, we aggregated effect estimates from individual genetic instruments using the IVW method. The IVW analysis indicated that genetically predicted zinc per standard-deviation increase was inversely associated with three types of epilepsy, encompassing all types of epilepsy (OR = 1.06, 95% CI: 1.02–1.11, *p* = 0.008), generalized epilepsy (OR = 1.13, 95% CI: 1.01–1.25, *p* = 0.030), and focal epilepsy (documented hippocampal sclerosis) (OR = 1.01, 95% CI: 1.00–1.02, *p* = 0.025) (Table 3). Sensitivity analysis indicated that none of the selected instruments exhibited horizontal pleiotropy (P intercept >0.05) or heterogeneity (P *Q* > 0.05) (Supplementary Table S6).

**Table 3:** IVW-MR association between zinc and epilepsy outcomes.

Two sample Mendelian randomization											
Outcome	Exposure	Method	NSNP	Beta	Se	CI lower bound	CI upper bound	OR	OR lower bound	OR upper bound	*p*-value
All epilepsy	Zinc	Inverse variance weighted	5	0.062	0.023	0.016	0.108	1.064	1.016	1.114	0.008*
		Weighted median	5	0.072	0.031	0.011	0.133	1.075	1.011	1.142	0.020*
		MR Egger	5	0.063	0.110	−0.153	0.279	1.065	0.858	1.322	0.608
Generalized epilepsy	Zinc	Inverse variance weighted	5	0.117	0.054	0.012	0.223	1.125	1.012	1.250	0.030*
		Weighted median	5	0.060	0.054	−0.046	0.165	1.061	0.955	1.180	0.268
		MR Egger	5	0.028	0.250	−0.461	0.518	1.029	0.630	1.679	0.917
Focal epilepsy	Zinc	Inverse variance weighted	5	0.037	0.027	−0.015	0.089	1.037	0.985	1.093	0.168
		Weighted median	5	0.039	0.033	−0.025	0.103	1.040	0.975	1.109	0.234
		MR Egger	6	0.038	0.108	−0.174	0.251	1.039	0.840	1.285	0.747
JME	Zinc	Inverse variance weighted	6	−0.004	0.006	−0.015	0.008	0.996	0.985	1.008	0.523
		Weighted median	6	−0.009	0.006	−0.021	0.003	0.991	0.979	1.003	0.127
		MR Egger	6	−0.033	0.018	−0.069	0.002	0.967	0.934	1.002	0.139
JAE	Zinc	Inverse variance weighted	6	0.005	0.003	−0.001	0.010	1.005	0.999	1.010	0.104
		Weighted median	6	0.006	0.004	−0.001	0.013	1.006	0.999	1.013	0.123
		MR Egger	6	0.004	0.010	−0.016	0.025	1.004	0.984	1.025	0.692
CAE	Zinc	Inverse variance weighted	6	0.007	0.004	−0.002	0.015	1.007	0.998	1.015	0.122
		Weighted median	6	0.005	0.005	−0.005	0.015	1.005	0.995	1.015	0.321
		MR Egger	6	−0.009	0.015	−0.039	0.022	0.991	0.962	1.022	0.606
Focal epilepsy (documented hippocampal sclerosis)	Zinc	Inverse variance weighted	6	0.009	0.004	0.001	0.018	1.009	1.001	1.018	0.025*
		Weighted median	6	0.012	0.005	0.003	0.022	1.013	1.003	1.022	0.010*
		MR Egger	6	0.032	0.013	0.006	0.057	1.032	1.006	1.059	0.073
Focal epilepsy (documented lesion negative)	Zinc	Inverse variance weighted	6	0.014	0.007	−0.001	0.029	1.014	0.999	1.029	0.060
		Weighted median	6	0.018	0.009	0.001	0.035	1.018	1.001	1.035	0.042*
		MR Egger	6	0.037	0.028	−0.018	0.091	1.037	0.982	1.095	0.258
Focal epilepsy (documented lesion other than hippocampal sclerosis)	Zinc	Inverse variance weighted	6	0.003	0.007	−0.009	0.016	1.003	0.991	1.016	0.596
		Weighted median	6	0.000	0.008	−0.017	0.017	1.000	0.984	1.017	0.996
		MR Egger	6	0.005	0.023	−0.041	0.051	1.005	0.960	1.052	0.842
Generalized epilepsy with tonic–clonic seizures	Zinc	Inverse variance weighted	6	0.000	0.002	−0.004	0.005	1.000	0.996	1.005	0.824
		Weighted median	6	0.000	0.003	−0.005	0.006	1.000	0.995	1.006	0.920
		MR Egger	6	0.000	0.008	−0.016	0.015	1.000	0.984	1.015	0.952

OR, odds ratio; CI, confidence interval.

## 4 Discussion

Grains and pulses are major zinc sources for the majority of people worldwide (28). In the United States, around 30% of dietary zinc is derived from pulses and cereals, with 50% coming from meat, and 20% from dairy products (29). Animal-based foods are the primary sources of easily accessible zinc. While fish and poultry have lower zinc content compared to red meat, which is the most abundant and common source of the mineral (30). Discussions typically focus on the potential adverse health effects associated with zinc intake, considering both deficiency (when intake is too low) and toxicity (when intake is too high). Whether diagnosing healthy populations or special populations, it is essential to investigate dietary zinc intake within the appropriate range. This ensures a comprehensive understanding of its impact on health outcomes, considering the varied dietary sources and potential adverse effects associated with both deficiency and toxicity.

We integrated NHANES 2013–2018 data, incorporating 4,434 US participants aged 20 or older in this study. The investigation assessed the association between dietary zinc intake and epilepsy, revealing an L-shaped relationship in the adult American population. The lowest risk of epilepsy was associated with a daily zinc intake of 5.0–11.0 mg. Stratified analyses affirmed the robustness of the relationship. Importantly, Mendelian randomization analyses indicated a potential causal link between zinc and certain types of epilepsy, including all types of epilepsy, generalized epilepsy, and focal epilepsy (documented hippocampal sclerosis). This study represents the initial exploration of the relationship between dietary zinc intake and epilepsy in the US adult population, providing insights into a potential causal connection.

Zinc, a crucial divalent cation and the second most abundant metal in the human body, plays an indispensable role in supporting life. Despite being required in minimal quantities, around 100 enzymes depend on zinc to execute vital chemical reactions. The potential link between zinc and epilepsy is substantiated by the observation that the highest levels of zinc in the brain resided in the hippocampus (31)—an essential region for cognition (32–34) and mood (35, 36), extensively studied in relation to epilepsy (37). Seizures, often associated with an imbalance between neuronal excitation and inhibition (38), reveal zinc’s interaction with both excitatory (glutamatergic) and inhibitory (γ-aminobutyric acid (GABA)-ergic) systems, influencing excitation and inhibition. Notably, zinc demonstrates dose-dependent actions, serving both pro- and anti-convulsant roles. Despite zinc’s diverse functions in the central nervous system, the concept of homeostasis succinctly captures its multifaceted roles (39, 40). Alterations in zinc homeostasis, whether through excessive or insufficient zinc intake, can disrupt cellular systems, highlighting the delicate balance required for optimal functioning.

Zinc, beneficial at normal levels, becomes detrimental when homeostasis is disrupted, leading to neuronal death through distinct mechanisms. In mice with epilepsy, a low zinc diet decreases brain zinc levels, increasing susceptibility to seizures (41), while a high zinc diet has the opposite effect. Zinc’s impact on brain function during seizures is dose-dependent, with levels above a certain threshold (>100 mM) potentially causing neural cell death (42). Studies reveal low calcium, iron, and zinc intake in children with refractory epilepsy, along with notably reduced serum zinc levels in individuals with epilepsy (43–45). Unchecked zinc supplementation can be toxic and induce epilepsy (3). Clinical trials show variations in serum zinc levels in epilepsy patients, suggesting a potential link between zinc and epilepsy (46). Moderate zinc intake, along with standard anti-epileptic medications, demonstrates a synergistic effect (41). Monitoring zinc levels and providing supplements as needed could serve as adjuvant therapies for epilepsy treatment. Our study aimed to evaluate dietary zinc intake in US adult epilepsy patients and analyze its impact on seizures.

Most nutritional epidemiology relies on food frequency questionnaires, but MR provides an alternative method to establish causal relationships. In this study, a two-sample MR analysis was employed to investigate the causal link between zinc and epilepsy. Our findings suggest a causal relationship between zinc and certain forms of epilepsy, including all types of epilepsy, generalized epilepsy, and focal epilepsy (documented hippocampal sclerosis). The robustness of our MR study is underscored by the extensive GWAS summary data and sensitivity analyses, confirming the resilience of our results. This method allowed us to mitigate confounding variables and potential biases in observational research. In summary, integrating GWAS-derived genetic risk factors with the MR design and IVW statistic method enhances the reliability of our findings, providing valuable insights into the relationship between zinc and epilepsy.

### 4.1 Limitations

However, our study has some limitations. One such limitation is the relatively small number of individuals with epilepsy included in the statistical analysis, a result of the NHANES database adopting ICD codes for diagnosis post-2013. Additionally, biological measures were absent in this study. Dietary data were collected using a 24 h memory test, relying on the participant’s ability to recall information, which may introduce recall bias and not accurately reflect typical intake. Due to the cross-sectional design, strict control over other influencing variables was not feasible. Despite employing genetic variants with strong associations as instrumental variables, the possibility of weak instrument bias remains. In our observational investigation, we identified a nonlinear association between zinc and epilepsy. However, nonlinear causality cannot be ruled out since the MR study only examined linear causal relationships. Moreover, our study predominantly includes individuals of European ancestry, limiting the generalizability of our findings to non-European populations. Additionally, the sample size for specific epilepsy subtypes was relatively small.

Although zinc levels may influence the development and control of seizures in individuals with epilepsy, it’s important to acknowledge that epilepsy itself has a diverse range of underlying causes. Various factors may contribute to its onset and severity, indicating that the influence of zinc is just one aspect among many. Therefore, while the findings of our study suggest a potential association between zinc and epilepsy, it’s essential to exercise caution in drawing definitive conclusions. Further research, including more comprehensive and detailed studies, is necessary to substantiate and expand upon our findings.

## 5 Conclusion

The results suggest that individuals with epilepsy experienced the lowest risk when their daily zinc intake ranged from 5.0 to 11.0 mg. From both clinical and nutritional perspectives, maintaining a moderate dietary zinc intake could offer benefits for people with epilepsy, potentially introducing a novel avenue for epilepsy treatment. Through the utilization of a 2-sample Mendelian randomization approach, we investigated the association between zinc and epilepsy, and our findings lend support to the notion that elevated zinc levels are associated with an increased risk of specific types of epilepsy. Nonetheless, it’s important to exercise caution in drawing definitive conclusions.

## Data availability statement

The original contributions presented in the study are included in the article/Supplementary material, further inquiries can be directed to the corresponding author.

## Ethics statement

The National Center for Health Statistics Ethics Review Committee granted ethics approval, and subjects gave signed informed consent. The studies were conducted in accordance with the local legislation and institutional requirements. Written informed consent for participation was not required from the participants or the participants’ legal guardians/next of kin in accordance with the national legislation and institutional requirements.

## Author contributions

SH: Data curation, Formal analysis, Investigation, Methodology, Software, Validation, Writing – original draft, Writing – review & editing. YG: Formal analysis, Methodology, Software, Writing – original draft, Writing – review & editing. YC: Data curation, Investigation, Project administration, Software, Writing – original draft. YW: Data curation, Resources, Software, Visualization, Writing – original draft. YL: Conceptualization, Investigation, Methodology, Software, Supervision, Writing – original draft. WG: Conceptualization, Data curation, Formal analysis, Investigation, Validation, Writing – review & editing. XH: Formal analysis, Funding acquisition, Investigation, Project administration, Visualization, Writing – original draft, Writing – review & editing. QF: Formal analysis, Funding acquisition, Project administration, Supervision, Visualization, Writing – original draft, Writing – review & editing.

## Footnotes

^1^ http://www.cdc.gov/nchs/nhanes.htm
