# Supplementary material for: Association between dietary zinc intake and epilepsy: findings from NHANES 2013–2018 and a Mendelian randomization study
Source: Front Nutr. 2024 Jul 10;11:1389338. doi: 10.3389/fnut.2024.1389338 (PMC11267886; doi:10.3389/fnut.2024.1389338)
Supplement: Supplementary file 1 [file Table_1.DOCX]

**Supplementary Material**

**Association between Dietary Zinc Intake and Epilepsy: Findings from NHANES 2013-2018 and a Mendelian Randomization Study**

**Catalogue:**

A description of the NHANES database (questionnaires, data analyzed, how was zinc intake estimated etc)

**Supplementary Table s1.** Frequency of each ASM with indication for treating seizures, counting towards our case definition of epilepsy.

**Supplementary Table s2.** Phenotype descriptions and distributions.

**Supplementary Table s3.** Information of genetic instrumental variants associated with zinc.

**Supplementary Table s4.** Threshold effect analysis of the relationship of zinc intake with epilepsy.

**Supplementary Table s5.** Subgroup analysis of the relationship between zinc intake and epilepsy.

**Supplementary Table s6.** IVW-MR sensitivity analysis between zinc and Epilepsy outcomes.

**A description of the NHANES database**

**Overview of NHANES**

NHANES is designed to collect information about the health and diet of people in the United States. These data are used to fulfill specific goals. The overall goals of NHANES are to: Estimate the number and percent of persons in the U.S. population and designated subgroups with selected diseases and risk factors; Monitor trends in the prevalence, awareness, treatment, and control of selected diseases; Monitor trends in risk behaviors and environmental exposures; Analyze risk factors for selected diseases; Study the relationship between diet, nutrition, and health; Explore emerging public health issues and new technologies; and Establish a national probability sample of genetic material for future genetic testing.

Each year, a nationally representative sample of the civilian, non-institutionalized U.S. population, all ages, is interviewed and examined. NHANES data are released in two year cycles. Oneyear estimates may be produced if there is a compelling public health need and if one year of data can provide a reliable estimate. Data from two year cycle NHANES 1999 through 2010 are posted on the NHANES website. The URL is http://www.cdc.gov/nchs/nhanes.htm. A major advantage of continuous NHANES data collection is the ability to address emerging public health issues and provide objective data on more health conditions and issues.

Westat has been contracted to conduct the study through 2014. Each year nearly 7,000 individuals of all ages in households across the United States are randomly selected to participate. The study respondents include whites/others as well as an oversample of blacks, Hispanics, and starting in 1-5 2011, Asians. The study design also includes a representative sample of these groups by age, sex, and income level. Older persons will also be oversampled.

Selected persons are invited to take part in the survey by being interviewed in their homes. Household interview data is collected via Computer Assisted Personal Interviewing (CAPI) and includes demographic, socioeconomic, dietary, and health-related questions. Upon completion of the interview, sample persons are asked to participate in a physical examination. The examination will be conducted in a specially equipped and designed Mobile Examination Center (MEC), consisting of four trailers. The MEC houses all of the state-of-the-art equipment for the physical exam and the tests conducted. The trailers are divided into rooms to assure the privacy of each study participant during the examination and interview. This examination includes a physical examination conducted by a physician and laboratory tests, X-rays, and other health measurements and interviews conducted by highly trained medical personnel. The household interviews and MEC exams combined will collect data in the following important health related areas: Cardiovascular disease; Diabetes mellitus; Oral health; Infectious diseases and immunization status; Obesity, growth and development; Dietary intake and behavior; Nutritional status; ν Osteoporosis; Physical activity; Mental health; Environmental exposures; and Other health-related topics.

**Measuring Guides for the Dietary Recall Interview**

The nutritional assessment component of the current NHANES includes a 24-hour dietary recall interview for participants of all ages. Dietary recall interviews are conducted in person by trained dietary interviewers fluent in Spanish and English. The setting of the interview is a private room in the Mobile Examination Center (MEC). Each MEC dietary interview room contains a standard set of measuring guides. These tools are used to help the respondent report the volume and dimensions of the food items consumed. They are not intended to represent any one particular food, but rather are designed to help respondents estimate portion sizes. This set of measuring guides is designed specifically for use in the current NHANES setting with a target population of non-institutionalized U.S. civilians. Whereas the tools are helpful in portion size estimation for a wide variety of foods, culturally sensitive precautions are warranted when applying these guides to other populations.

In order to obtain a more complete picture of the usual dietary intake of the U.S. population, a second dietary interview for all participants who complete the in-person recall was added to the survey in 2002. The second dietary recall is collected by telephone and is scheduled 3 to 10 days later. Upon completion of the in-person interview, participants are given measuring cups, spoons, a ruler, and a food model booklet, which contain two-dimensional drawings of the various measuring guides available in the MEC, to use for reporting food amounts during the telephone interview.

**Table S1.** Frequency of each ASM with indication for treating seizures, counting towards our case definition of epilepsy.

| Antiseizure medication | Frequency ^a^ |
| --- | --- |
| Phenytoin | 19 |
| Levetiracetam | 15 |
| Lamotrigine | 11 |
| Gabapentin | 11 |
| Divalproex | 7 |
| Carbamazepine | 6 |
| Oxcarbazepine | 4 |
| Topiramate | 4 |
| Phenobarbital | 4 |
| Clonazepam | 2 |
| Primidone | 2 |
| Diazepam | 1 |
| Alprazolam | 1 |
| Pregabalin | 1 |
| Clobazam | 1 |

^a^ The sum (89) is greater than our sample size of participants treated for seizures (67) because some participants reported taking more than 1 ASM.

**Table S2.** Phenotype descriptions and distributions.

| PHENOTYPES | Consortium | First Author (Year) | Sample Size | N Cases | N Controls | Population | MRC-IEU ID: |
| --- | --- | --- | --- | --- | --- | --- | --- |
| Zinc | NA | Evans（2013） | 2603 | NA | NA | European | ieu-a-1079 |
| All Epilepsy | ILAE | Abou-Khalil B（2018） | 44889 | 15212 | 29677 | Mixed | ieu-b-8 |
| Generalized Epilepsy | ILAE | Abou-Khalil B（2018） | 33446 | 3769 | 29677 | Mixed | ieu-b-9 |
| Focal Epilepsy | ILAE | Abou-Khalil B（2018） | 39348 | 9671 | 29677 | Mixed | ieu-b-10 |
| JME | ILAE | Abou-Khalil B（2018） | 30858 | 1181 | 29677 | Mixed | ieu-b-17 |
| JAE | ILAE | Abou-Khalil B（2018） | 30092 | 415 | 29677 | Mixed | ieu-b-12 |
| CAE | ILAE | Abou-Khalil B（2018） | 30470 | 793 | 29677 | Mixed | ieu-b-13 |
| Focal Epilepsy (documented hippocampal sclerosis) | ILAE | Abou-Khalil B（2018） | 30480 | 803 | 29677 | Mixed | ieu-b-14 |
| Focal Epilepsy (documented lesion negative) | ILAE | Abou-Khalil B（2018） | 32393 | 2716 | 29677 | Mixed | ieu-b-11 |
| Focal Epilepsy (documented lesion other than hippocampal sclerosis) | ILAE | Abou-Khalil B（2018） | 32,747 | 3,070 | 29,677 | Mixed | ieu-b-15 |
| Generalized Epilepsy with Tonic-clonic Seizures | ILAE | Abou-Khalil B（2018） | 29905 | 228 | 29677 | Mixed | ieu-b-16 |

**Table S3.** Information of genetic instrumental variants associated with zinc.

| SNP | Chromosome | Position | Beta | SE | *P* | Effect Allele | Other Allele | Effect Allele Frequency | Sample size | R2 | F |
| --- | --- | --- | --- | --- | --- | --- | --- | --- | --- | --- | --- |
| rs10484100 | 14 | 86817096 | -0.209 | 0.045 | 3.30E-06 | G | A | 0.125 | 2603 | 8.22E-03 | 21.554 |
| rs10931753 | 2 | 154568757 | -0.129 | 0.028 | 4.94E-06 | C | G | 0.352 | 2603 | 8.09E-03 | 21.210 |
| rs11232535 | 11 | 80929352 | 0.325 | 0.065 | 6.73E-07 | C | T | 0.160 | 2603 | 9.51E-03 | 24.981 |
| rs11763353 | 7 | 15630871 | -0.192 | 0.039 | 6.90E-07 | G | A | 0.197 | 2603 | 9.23E-03 | 24.218 |
| rs1532423 | 8 | 86268313 | -0.178 | 0.026 | 6.40E-12 | G | A | 0.379 | 2603 | 1.77E-02 | 46.834 |
| rs7148590 | 14 | 65473196 | -0.140 | 0.026 | 1.37E-07 | A | G | 0.431 | 2603 | 1.10E-02 | 28.972 |

**Table S4.** Threshold effect analysis of the relationship of zinc intake with epilepsy.

| Zinc Intake mg/day | Adjusted Model | |
| --- | --- | --- |
|  | OR (95% CI) | *p*-value |
| <8.0 | 0.81 (0.67~0.98) | 0.031 |
| *≥*8.0 | 1.04 (0.97~1.11) | 0.252 |
| Likelihood Ratio test | - | 0.026 |

OR, odds ratio; CI, confidence interval. Adjusted for sociodemographic (age, sex, marital status, race/ethnicity, education level, family income) and dietary supplements taken. Only 99% of the data is displayed.

**Table S5.** Subgroup analysis of the relationship between zinc intake and epilepsy.

|  | T1 (≤5.0) | T2 (5.0-11.0) | T3 (≥11.0) | P for interaction |
| --- | --- | --- | --- | --- |
| Sex |  |  |  | 0.196 |
| Male | 1(Ref) | 0.56 (0.21~1.51) | 0.42 (0.15~1.16) |  |
| Female | 1(Ref) | 0.44 (0.19~1.01) | 0.93 (0.40~2.18) |  |
| Age, y |  |  |  | 0.844 |
| 20-50 | 1(Ref) | 0.33 (0.11~1.01) | 0.44 (0.14~1.35) |  |
| ＞50 | 1(Ref) | 0.57 (0.27~1.24) | 0.70 (0.30~1.61) |  |
| Marital status |  |  |  | 0.530 |
| Married or living with partner | 1(Ref) | 0.53 (0.19~1.49) | 0.48 (0.16~1.50) |  |
| Living alone | 1(Ref) | 0.45 (0.20~1.00) | 0.69 (0.30~1.57) |  |
| Education level, y |  |  |  | 0.955 |
| ≤12 | 1(Ref) | 0.43 (0.18~1.01) | 0.51 (0.21~1.27) |  |
| ＞12 | 1(Ref) | 0.55 (0.21~1.41) | 0.70 (0.26~1.91) |  |
| Family income |  |  |  | 0.286 |
| Low | 1(Ref) | 0.68 (0.28~1.66) | 1.14 (0.45~2.88) |  |
| Medium or high | 1(Ref) | 0.35 (0.14~0.84) | 0.31 (0.12~0.78) |  |
| Dietary supplements |  |  |  | 0.428 |
| Use of supplements | 1(Ref) | 0.85 (0.30~2.47) | 0.77 (0.24~2.43) |  |
| Without supplements | 1(Ref) | 0.35 (0.16~0.78) | 0.56 (0.25~1.26) |  |

T, tertiles; Ref: reference.

**Table S6.** IVW-MR sensitivity analysis between zinc and Epilepsy outcomes.

|  |  |  | Heterogeneity | | | Pleiotropy | | |
| --- | --- | --- | --- | --- | --- | --- | --- | --- |
| OUTCOME | EXPOSURE | METHOD | Q | Q DF | Q *P*VALUE | EGGER INTERCEPT | EGGER SE | EGGER *P* VALUE |
| All epilepsy | Zinc | Inverse variance weighted | 4.266 | 4 | 0.371 |  |  |  |
|  |  | MR Egger | 4.266 | 3 | 0.234 | 0.000 | 0.021 | 0.993 |
| Generalized Epilepsy | Zinc | Inverse variance weighted | 7.772 | 4 | 0.100 |  |  |  |
|  |  | MR Egger | 7.438 | 3 | 0.059 | 0.017 | 0.047 | 0.738 |
| Focal Epilepsy | Zinc | Inverse variance weighted | 2.737 | 4 | 0.603 |  |  |  |
|  |  | MR Egger | 2.737 | 3 | 0.434 | 0.000 | 0.020 | 0.988 |
| JME | Zinc | Inverse variance weighted | 8.111 | 5 | 0.150 |  |  |  |
|  |  | MR Egger | 4.698 | 4 | 0.320 | 0.006 | 0.004 | 0.163 |
| JAE | Zinc | Inverse variance weighted | 1.707 | 5 | 0.888 |  |  |  |
|  |  | MR Egger | 1.707 | 4 | 0.790 | 0.000 | 0.002 | 0.980 |
| CAE | Zinc | Inverse variance weighted | 6.159 | 5 | 0.291 |  |  |  |
|  |  | MR Egger | 4.865 | 4 | 0.301 | 0.003 | 0.003 | 0.361 |
| Focal Epilepsy (documented hippocampal sclerosis) | Zinc | Inverse variance weighted | 6.697 | 5 | 0.244 |  |  |  |
|  |  | MR Egger | 3.578 | 4 | 0.466 | -0.005 | 0.003 | 0.152 |
| Focal Epilepsy (documented lesion negative) | Zinc | Inverse variance weighted | 6.730 | 5 | 0.242 |  |  |  |
|  |  | MR Egger | 5.717 | 4 | 0.221 | -0.005 | 0.006 | 0.447 |
| Focal Epilepsy (documented lesion other than hippocampal sclerosis) | Zinc | Inverse variance weighted | 2.926 | 5 | 0.711 |  |  |  |
|  |  | MR Egger | 2.921 | 4 | 0.571 | 0.000 | 0.005 | 0.949 |
| Generalized Epilepsy with Tonic-clonic Seizures | Zinc | Inverse variance weighted | 3.383 | 5 | 0.641 |  |  |  |
|  |  | MR Egger | 3.366 | 4 | 0.499 | 0.000 | 0.002 | 0.903 |
