# Supplementary figures and images for: Association between dietary zinc intake and epilepsy: findings from NHANES 2013–2018 and a Mendelian randomization study
Source: Front Nutr. 2024 Jul 10;11:1389338. doi: 10.3389/fnut.2024.1389338 (PMC11267886; doi:10.3389/fnut.2024.1389338)

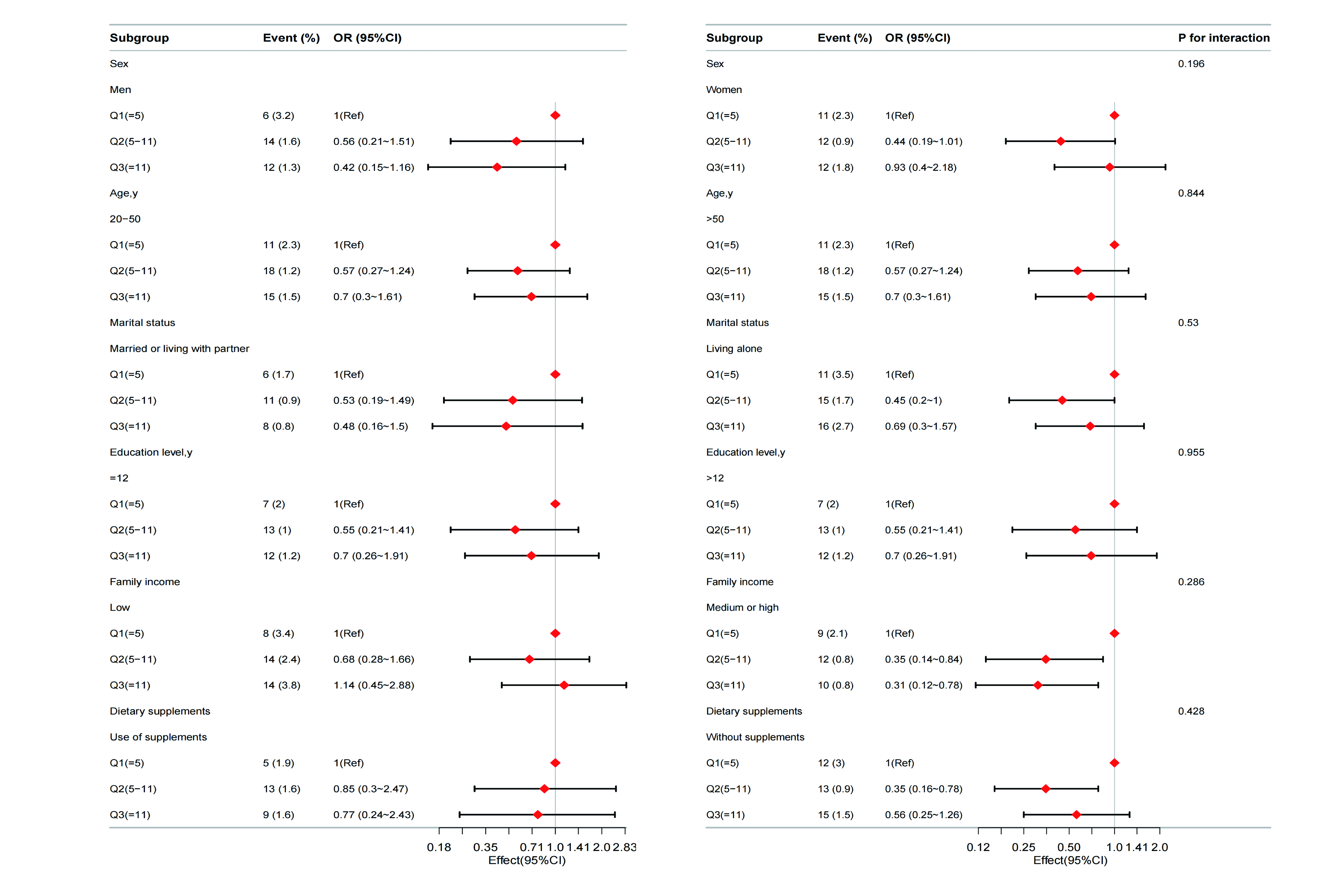

Supplement: Supplementary file 2 [file Image_1.TIF]
